# Supplementary material for: Disentangling metabolic functions of bacteria in the honey bee gut
Source: PLoS Biol. 2017 Dec 12;15(12):e2003467. doi: 10.1371/journal.pbio.2003467 (PMC5726620; doi:10.1371/journal.pbio.2003467)
Supplement: S8 Data — (ZIP) [file pbio.2003467.s008.zip › Z-score_plots/648 microbial product 2,3-Dinor-8-iso prostaglandin F2alpha.pdf]

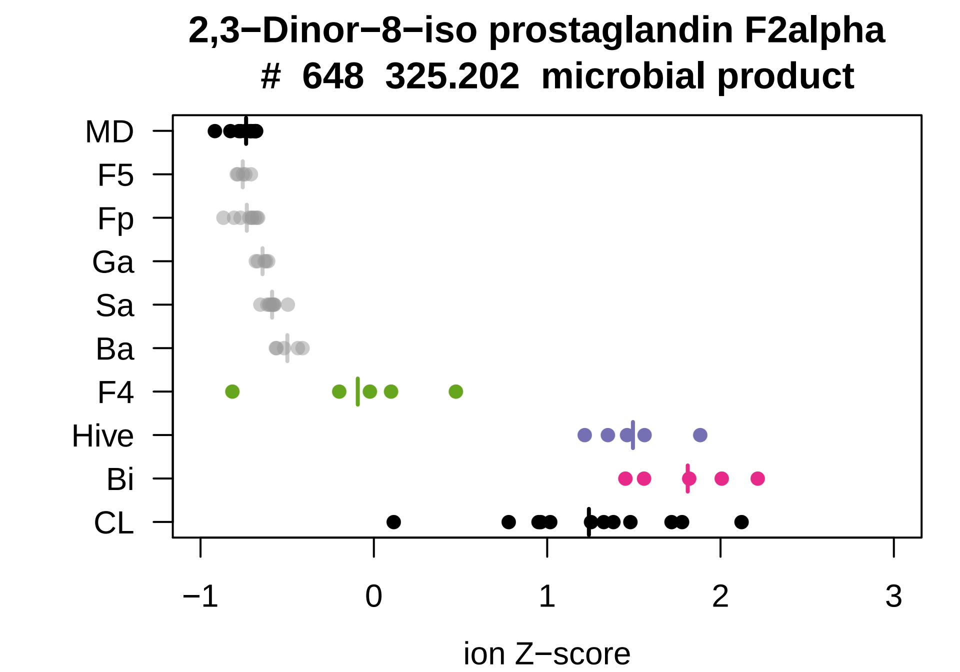

2,3−Dinor−8−iso prostaglandin F2alpha
648 325.202 microbial product
MD
F5
Fp
Ga
Sa
Ba
F4
Hive
Bi
CL
−1
0
1
2
3
ion Z−score
